# Identification of potential biomarkers from amino acid transporter in the activation of hepatic stellate cells via bioinformatics

**DOI:** 10.3389/fgene.2024.1499915

**Published:** 2024-12-04

**Authors:** Yingying Zhao, Xueqing Xu, Huaiyang Cai, Wenhong Wu, Yingwei Wang, Cheng Huang, Heping Qin, Shuangyang Mo

**Affiliations:** ^1^ Shandong University of Traditional Chinese Medicine, Jinan, China; ^2^ Liuzhou People's Hospital Affiliated to Guangxi Medical University, Liuzhou, China

**Keywords:** liver fibrosis, hepatic stellate cells, amino acid transporter, solute carrier family 7 member 5 (SLC7A5), solute carrier family 1 member 5 (SLC1A5), bioinformatics

## Abstract

**Background:**

The etiopathogenesis of hepatic stellate cells (HSC) activation has yet to be completely comprehended, and there has been broad concern about the interplay between amino acid transporter and cell proliferation. This study proposed exploring the molecular mechanism from amino acid transport-related genes in HSC activation by bioinformatic methods, seeking to identify the potentially crucial biomarkers.

**Methods:**

GSE68000, the mRNA expression profile dataset of activated HSC, was applied as the training dataset, and GSE67664 as the validation dataset. Differently expressed amino acid transport-related genes (DEAATGs), GO, DO, and KEGG analyses were utilized. We applied the protein-protein interaction analysis and machine learning of LASSO and random forests to identify the target genes. Moreover, single-gene GESA was executed to investigate the potential functions of target genes via the KEGG pathway terms. Then, a ceRNA network and a drug-gene interaction network were constructed. Ultimately, correlation analysis was explored between target genes and collagen alpha I (COL1A), alpha-smooth muscle actin (α-SMA), and immune checkpoints.

**Results:**

We identified 15 DEAATGs, whose enrichment analyses indicated that they were primarily enriched in the transport and metabolic process of amino acids. Moreover, two target genes (SLC7A5 and SLC1A5) were recognized from the PPI network and machine learning, confirmed through the validation dataset. Then single-gene GESA analysis revealed that SLC7A5 and SLC1A5 had a significant positive correlation to ECM−receptor interaction, cell cycle, and TGF−β signaling pathway and negative association with retinol metabolism conversely. Furthermore, the mRNA expression of target genes was closely correlated with the COL1A and α-SMA, as well as immune checkpoints. Additionally, 12 potential therapeutic drugs were in the drug-gene interaction network, and the ceRNA network was constructed and visualized.

**Conclusion:**

SLC7A5 and SLC1A5, with their relevant molecules, could be potentially vital biomarkers for the activation of HSC.

## Introduction

Liver fibrosis is the pathologic reaction to persistent hepatic injury triggered by various etiological factors, which can directly lead to the ultimate consequences of liver cirrhosis and end-stage liver failure, placing a threat to public health worldwide (Ogaly et al., 2018). It was reported that liver fibrosis is responsible for a large number of clinical cases and deaths annually (Luo et al., 2021). Hepatic stellate cells (HSC), situated in the space of Disse, are essential mesenchymal cells and perform a crucial function for liver physiology. Usually, HSC are in a quiescent state and store retinol (Yang et al., 2019). The activation of HSC is considered the main event and promoter of liver fibrosis. The migration, proliferation, and differentiation of activated HSC (aHSC) can stimulate the excessive cumulation of extracellular matrixes (ECM) with retinol loss (Khazali et al., 2018). However, with recent advances in the awareness of HSC activation and their dominant position in liver fibrosis, the particular molecular mechanisms of regulating these processes are unclear.

Energy metabolism is the foundation of cell proliferation, biosynthesis, and physiological activity (Zhang et al., 2017). Rapidly proliferating cells utilize a diversity of strategies for metabolism to fulfill the energetic demands of cell growth, biosynthesis, and karyokinesis (Black et al., 2020). This shift has been defined as metabolic reprogramming, which is a hallmark of various diseases (Rodenfels et al., 2019). In reaction to harm factors, HSC are activated, transdifferentiating from a quiescent form to a myofibroblast state characterized by proliferation, migratory, invasive capabilities, and excessive production of ECM. HSC need to exert a diversity of metabolic energy strategies to meet the extraordinary requirements for exuberant biosynthesis and proliferation (Gu et al., 2019). A study demonstrated that the metabolic reprogramming of glycogen, ascorbic acid, and amino acids metabolism active remodeling of the ECM, proceeding to liver fibrogenesis in reaction to persistent impairment (Nault et al., 2016). Du et al. illustrated that the hepatic glutamine uptake in liver fibrosis models of mice induced with CCl4 was significantly higher compared with control mice, and suppression of glutaminolysis was proved to prevent the accumulation of fibrogenesis (Chen et al., 2012). Glutamine is an essential substrate of metabolic reprogramming in many cancer cells, which can facilitate rapid proliferation and biosynthesis (Du et al., 2020). Furthermore, the Hedgehog-YAP signaling pathway may correlate closely to the HSC activation through regulating glutaminolysis (Du et al., 2018). Similarly, Leucine could promote the biosynthesis of collagen alpha I (COL1A) in HSC by stimulating the regulative translation mechanisms and PI3K/Akt/mTOR signaling pathways (Pérez de Obanos et al., 2006). Along these lines, regulating the cellular amino metabolism of HSC may represent a potential fresh therapeutical target for liver fibrogenesis.

Amino acids are categorized into non-essential and essential types, with the latter not being synthesized from scratch. In mammals, various amino acid transporters (AAT) located in the plasma membrane or intracellular compartments, such as the Golgi apparatus, lysosomes, and mitochondria, contribute to the regulation of amino acids transmembrane transport and promote the transmembrane exchange of other substrates (Zhang et al., 2020). Some studies indicated that regulating the amount or activity of particular AAT may contribute to modulating the proliferation of eukaryotes; moreover, AAT are closely correlated with cancer patients’ prognosis, metastasis, and survival (Lin et al., 2020). The solute carrier family 7 member 5 (SLC7A5; LAT1), which especially participates in the transport of large neutral amino acids, is extraordinarily upregulated in hepatocellular carcinoma, and suppression of SLC7A5 leads to downregulates global translation in cancer cells (Li et al., 2013). The amino transporter SLC6A14, regulating the transmembrane uptake of amino acids, is upregulated in many human cancers characterized by a growing requirement for amino acids (Yazawa et al., 2015). Analogously, the expression of SLC38A1 in colorectal carcinoma is closely relevant to the clinical stage of tumor node metastasis (TNM). The SLC38A1 downregulation can restrain tumor expansion and inhibit the migration of colorectal cancer cells (Zhou et al., 2017). The alterations of metabolism that appeared in HSC activation share various customary characteristics with cancer cells (Gauthier-Coles et al., 2021). Thus, we hypothesized that modulation of amino acid metabolic by AAT could have a role in aHSC operation. However, our understanding of the specific relationship between AAT and the activation of HSC is limited.

In contemporary life science research, the advancement of high-throughput sequencing and microarray technologies has positioned bioinformatics as an essential tool. It is employed to analyze differentially expressed mRNA and to predict potential therapeutic targets for specific diseases. Bioinformatic analysis is an efficacious approach to discovering biomarkers of etiopathogenesis of ailments and provides an estimable foundation for further studies (Peng S. et al., 2022). Consequently, this study involved an analysis of datasets from the Gene Expression Omnibus (GEO) (Clough and Barrett, 2016) via bioinformatic methods to determine the molecular mechanism of amino acid transport-related genes in activating HSC and recognized critical biomarkers. Furthermore, a drug-gene interaction meshwork and ceRNA network were established.

## Material and method

### Microarray data source

**FIGURE 1:**
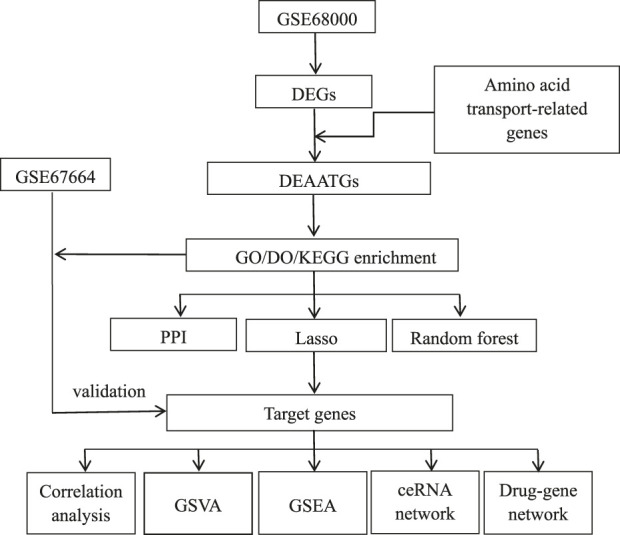
Flowchart of the study.

Figure 1 illustrates the analysis procedure for this research. Data series GSE68000 and GSE67664 were downloaded from the GEO database. There were 11 samples’ mRNA expression profiling of GSE68000, including 3 aHSC and 3 qHSC samples. GSE67664 contained 13 samples, including 4 aHSC and 4 qHSC samples. Further information can be found in Table 1. Dataserie GSE68000 was the training set, and data series GSE67664 was the validation set.

**TABLE 1:** Details of the GEO data.

Dataset	Platform	Number of samples (activated/quiescent HSC, subjects)
GSE68000	GPL13667 [HG-U219] Affymetrix Human Genome U219 Array	11 (3/3,6)
GSE67664	GPL19099 [HG-U219] Affymetrix Human Genome U219 Array	13 (4/4,8)

GEO, gene expression omnibus.

### Identifying differently expressed amino acid transport-related genes

Differentially expressed genes (DEGs) between the aHSC and qHSC were identified using normalized data processed with the GEO2R tool (Barrett et al., 2013), applying a threshold of |log2 Fold Change| >1 and p < 0.05. The GeneCards Database was used to download 33 genes involved in the amino acid transport pathway across the plasma membrane (https://pathcards.genecards.org/Pathway/3132). Altogether, identical genes in DEGs and amino acid transport across the plasma membrane pathway were defined as differently expressed amino acid transport-related Genes (DEAATGs).

### GO, DO, and KEGG enrichment analyses

Gene ontology (GO) enrichment [included biological process (BP), cellular component (CC), and molecular function (MF)] analysis and Kyoto Encyclopedia of Genes and Genomes (KEGG) pathway analysis were applied by utilizing the R clusterProfiler package (Yu et al., 2012). The false discovery rate (FDR) was calculated via Benjamini–Hochberg (BH) adjustment. The cutoff criterion was q-value <0.05. We utilized the R DOSE package to apply the enrichment analysis of Disease Ontology (DO) terms (Yu et al., 2015). Ultimately, the significant outcomes of these enrichment analyses were visualized using the R ggplot2 and pathview packages.

### Investigating protein-protein interaction networks and hub genes

We predicted and constructed a protein-protein interaction (PPI) network using the STRING database by uploading DEAATGs. The network was visualized and analyzed with Cytoscape 3.9.1, and hub genes were identified using the cytohubba plugin.

### LASSO and random forest

Subsequently, machine learning techniques, including LASSO regression and the random forests (RF) algorithm, were employed to identify feature genes from DEAATGs. The LASSO regression model was optimized by determining the optimal parameter λ through 10-fold cross-validation, configured with “family = binomial” and “measure = deviance,” while all other parameters were set to their default values (Engebretsen and Bohlin, 2019). Concurrently, the RF method was applied to differentiate feature genes from DEAATGs utilizing the R randomforest package (Pavey et al., 2017). Within the RF algorithm, which has a feature selection capability, the MeanDecreaseGini value signifies the importance of a feature. Each input gene of DEAATGs was ranked by order of importance in the classification using their MeanDecreaseGini score. Genes with a MeanDecreaseGini score not equal to zero were identified as feature genes of the RF model.

### Target genes recognition

In this study, we identified the consistently present genes within the hub and feature gene set of the two aforementioned machine-learning models as target genes.

### Data verification of target genes

The validation set was derived from the dataset GSE67664, comprising 4 aHSC samples and 4 qHSC samples, and was utilized to verify the reliability of these target genes.

### Gene Set Enrichment Analysis and Gene Set Variation Analysis

We investigated the roles of target genes in HSC activation by conducting single-gene Gene Set Enrichment Analysis (GSEA) using the R clusterProfiler package. Each target gene’s expression level was used to categorize samples into low- and high-expression groups. GSEA was then applied to identify significantly different KEGG pathways between these groups. Then a nonparametric unsupervised method of Gene Set Variation Analysis (GSVA) was performed to demonstrate the differential enrichment KEGG pathways between the two groups similarly. In this study, the R GSVA package was utilized with the gene sets of c2.cp.kegg.symbols.gmt, downloaded from the official site. A p-value of less than 0.05 was established as the threshold for statistical significance.

### Investigation of ceRNA network of target genes

The Prospective miRNAs associating the target genes were predicted via the miRTarBase (Huang et al., 2022), TargetScan (McGeary et al., 2019), and Starbase (Li et al., 2014), aiming at exploring the mRNA–miRNA interaction of the ceRNA network. A miRNA identified simultaneously in all three databases was enrolled, and then the potential lncRNAs targeting the miRNA were recognized from the spongeScan website (http://spongescan.rc.ufl.edu/). Ultimately, the data visualization with a Venn diagram was performed through a web-based tool (https://www.bioinformatics.com.cn/), and the ceRNA network was visualized with Cytoscape 3.9.1 software.

### Drug–gene interaction meshwork

The DrugBank database was employed to predict current or potentially related drug substances for studying the drug-gene link (Wishart et al., 2018). Moreover, Cytoscape software was used to construct the data visualization of the drug-gene interaction network.

### Identification of protein subcellular localization

We used the Cell-PLoc 2.0 tool (Chou and Shen, 2008), a package from a website, to predict the subcellular localization of proteins coded by target genes.

### Assessment of the correlation with immune checkpoints

The correlation between the target gene and vital immune checkpoints (Liu, 2019), such as PD1, CTLA4, LAG3, TIGIT, HAVCR2, and PDL1, was studied with Pearson’s correlation coefficient. Then the website tool (http://www.bioinformatics.com.cn/) was utilized for data visualization with a scatter diagram.

### Analysis of correlation with common key biomarkers of activated HSC

α-SMA (ACTA2) and COL1A was considered the key biomarkers for activation of HSC to a fibrogenic myofibroblast. Similarly, the correlation analyses between the target genes and these key biomarkers in HSC activation were proceeded by Pearson’s correlation coefficient.

### Statistical analysis

A Student’s t-test for unpaired samples was conducted to analyze the data between the two groups, and Pearson’s correlation coefficient was utilized for the correlation analysis between the two variables. p < 0.05 was set as the cutoff.

## Result

### Recognition of DEGs

**FIGURE 2:**
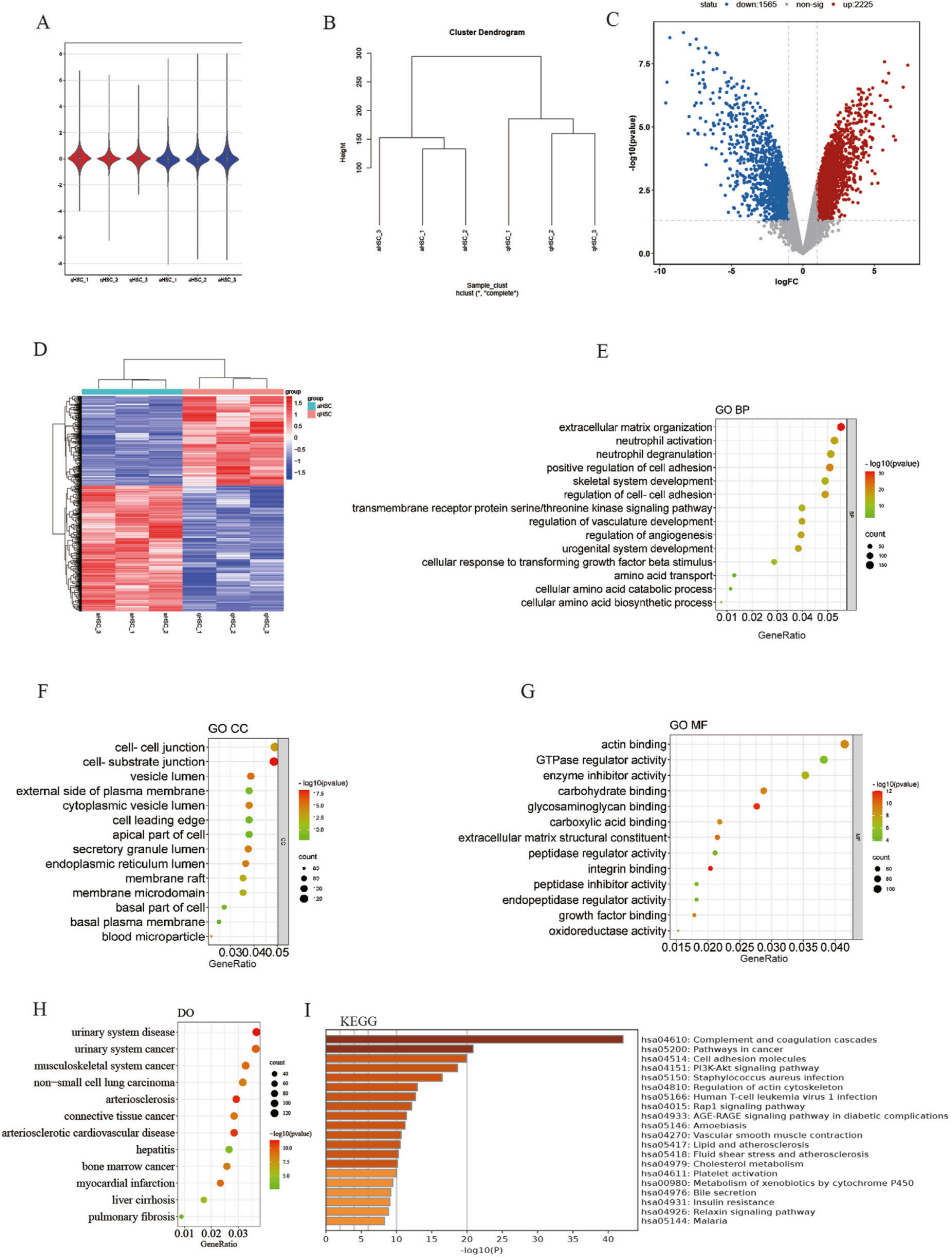
Identification of DEGs and erichment analyses. **(A)** GSE68000 data after normalization; **(B)** Sample cluster of GES68000; **(C)** The volcano plot of the GSE68000; **(D)** The heatmap ofDEGs; **(E)** GO BP; **(F)** GO CC; **(G)** GO MF; **(H)** DO enrichment; **(I)** KEGG signaling pathway.

The mRNA expression profile dataset (GSE68000) for HSC was normalized, as depicted in Figures 2A, B. Subsequently, 3,775 DEGs were identified from the GSE68000 dataset, comprising 2,225 upregulated and 1,565 downregulated DEGs. A volcano plot and heatmap illustrating these findings are presented in Figures 2C, D, respectively.

### Enrichment analyses of the DEGs

The GO and DO analyses of the DEGs were conducted to elucidate their potential biological functions. Within the GO BP cluster, the DEGs are predominantly involved in the regulation of extracellular matrix organization, positive regulation of cell adhesion, amino acid transport, cellular amino acid biosynthesis process, etc. (Figure 2E). The majority of DEGs are found in the membrane microdomain, cell-cell junction, and basal plasma membrane within the GO CC cluster (Figure 2F). The GO MF cluster shows that mainly DEGs were enriched in actin binding, GTPase regulator activities, glycosaminoglycan binding, ECM structural constituent and growth factor binding, etc. (Figure 2G). In the DO category, the DEGs mainly participated in the malignant tumor, liver cirrhosis, pulmonary fibrosis, etc. (Figure 2H). Finally, the outcomes from KEGG pathway enrichment showed that the DEGs mostly participated in cancer pathways, cell adhesion molecules, PI3K-Akt signaling pathway, and cholesterol metabolism. (Figure 2I).

### Recognition of DEAATGs

**FIGURE 3:**
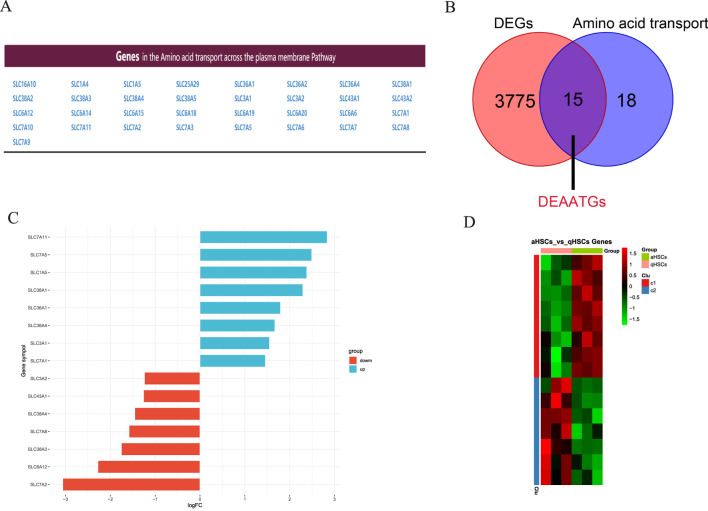
Identification of DEAATGs. **(A)** Amino acid transport-related genes; **(B)** The DEAATGs of GES68000; **(C)** The details of each DEAATGs; **(D)** The heatmap of DEATTGs (Clu: Cluster).

Figure 3A shows the genes involved in the pathway for transporting amino acids across the plasma membrane. Then we utilized an integrated bioinformatics analysis to identify a total of 15 congruent DEAATGs, containing 8 genes consistently upregulated and 7 genes congruously downregulated (Figures 3B, C; Table 2). The heatmap for DEAATGs is exhibited in Figure 3D.

**TABLE 2:** The DEAATGs of GSE68000.

Regulation	DEAATGs
Upregulated (n = 8)	SLC1A5, SLC38A1, SLC7A5, SLC36A1, SLC38A1, SLC36A4, SLC3A1, SLC7A1
Downregulated (n = 7)	SLC7A11, SLC7A8, SLC7A2, SLC6A12, SLC43A1, SLC38A3, SLC3A2

DEAATGs, differently expressed amino acid transport-related genes.

### Function enrichment analyses of the DEAATGs

**FIGURE 4:**
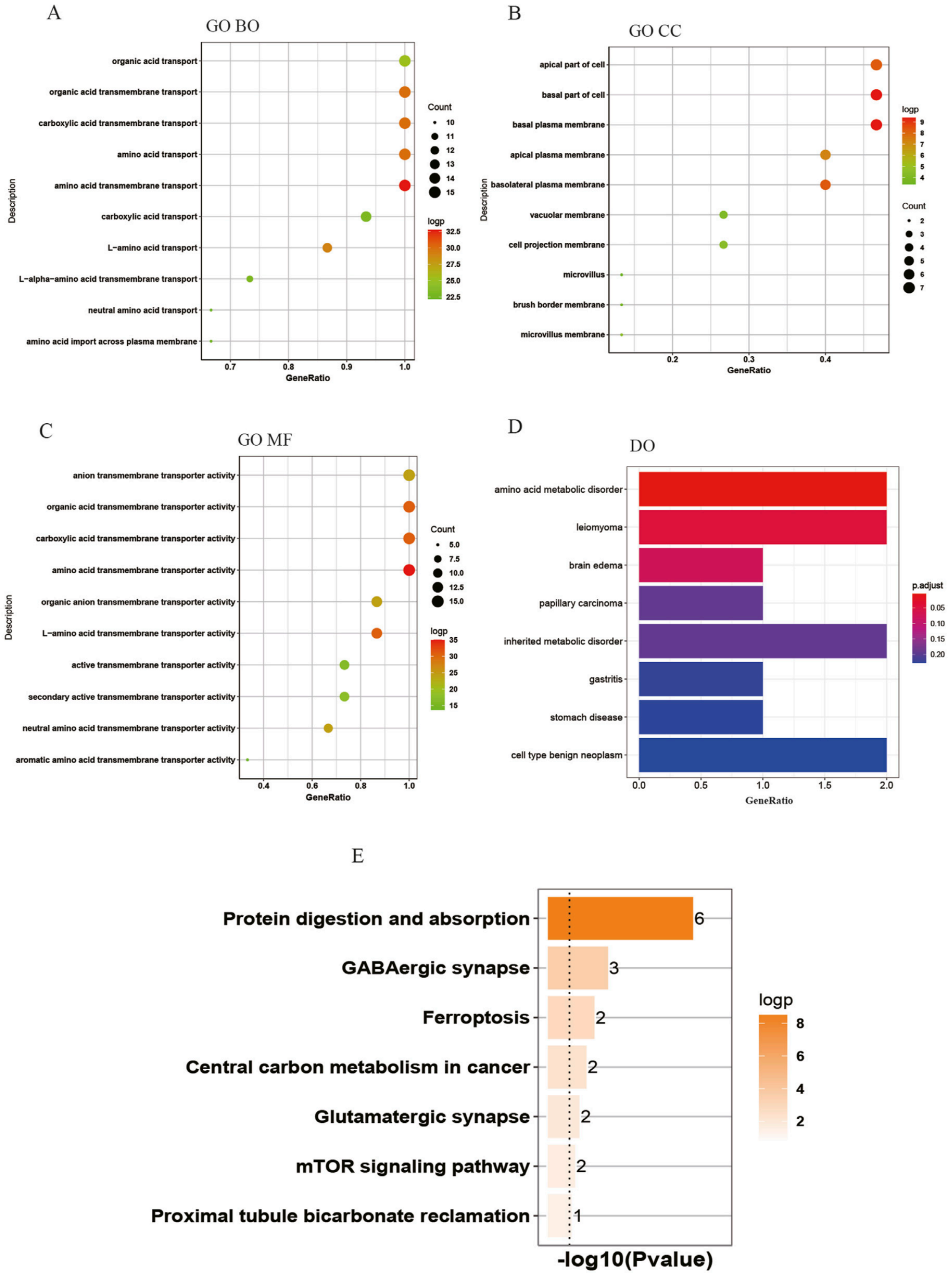
Enrichment analyses of DEAATGs. **(A)** GO BP; **(B)** GP CC; **(C)** GO MF; **(D)** DO enrichment; **(E)** KEGG signaling pathway.

The biology functions of DEAATGs were performed in GO and DO analyses. Expectedly, the DEAATGs were mainly located in the cell membranes and promoted the transport of various amino acids across the plasma membrane (Figures 4A, B). In the GO MF category, the DEAATGs contribute particularly to regulating the cellular metabolic activity of plentiful amino acids. (Figure 4C). The R package DOSE was utilized to conduct a comprehensive investigation into the function of DEAATGs. The findings from the DO enrichment analysis indicated that DEAATGs may be involved in amino acid metabolic disorder, inherited metabolic disorder, leiomyoma, cell type benign neoplasm, etc., which were the principal diseases (Figure 4D). KEGG pathway enrichment analysis indicates that DEAATGs are primarily linked to protein digestion and absorption, ferroptosis, central carbon metabolism in cancer, and the mTOR signaling pathway (Figure 4E). These findings imply that DEAATGs primarily function in regulating cell proliferation, ferroptosis, and cellular metabolism, especially amino acid transport.

### PPI network and hub gene analyses

**FIGURE 5:**
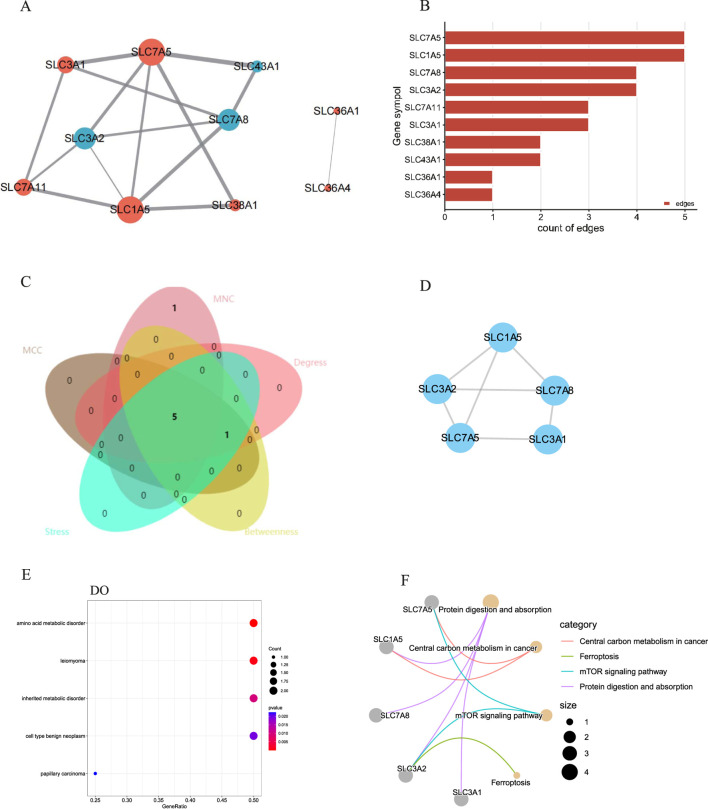
The PPI network and hub gene analyses. **(A)** The PPI network of the DEAATGs, the bigger sizes of the edge and node mean the higher degree,The red means upregulated, and blue means downregulated; **(B)** The connectivity rank of genes; **(C)** Five algorithms were utilized to identified hub genes and venn diagram; **(D)** The PPI network of hub genes; **(E)** DO enrichment of hub genes; **(F)** KEGG enrichment of hub genes.

A PPI network of the DEAATGs was built using the STRING database to investigate the relationship of each protein, consisting of 10 nodes and 15 edges. In this PPI network map, each node represented a protein, and simultaneously each edge represented an association between two proteins. Additionally, within the 10 nodes, 7 nodes were upregulated, and 3 were downregulated (Figure 5A). As displayed in Figure 5B, **T**he genes were ranked by target connectedness from large to small within the PPI network graph. Hub genes were identified congruously through five categories (degree, betweenness, MNC, MCC, and stress) from cytoHubba Plug-in of Cytoscape software. Finally, we extracted the intersection of the top 6 hub genes and screened out 5 hub genes, SLC7A5, SLC7A8, SLC1A5, SLC3A2, and SLC3A1 might have a crucial function in the PPI network (Figures 5C, D; Table 3). The outputs of DO enrichment and KEGG pathway enrichment of these 5 hub genes are shown in Figures 5E, F. They still primarily enrich in the mTOR signaling pathway, ferroptosis, and cellular metabolism of amino acids.

**TABLE 3:** The top five hub genes.

Genes	MCC	MNC	Degree	Stress	Betweenness	Log_2_FC
SLC7A5	6	3	5	22	10.3	2.143191791
SLC1A5	8	5	5	16	7.5	2.382896913
SLC7A8	4	2	4	16	5.3	1.580877908
SLC3A1	3	2	3	4	1.3	1.550995367
SLC3A2	4	2	4	16	5.3	-1.237840961

MCC, maximal clique centrality; MNC, maximum neighborhood component.

### The machine learning algorithm of LASSO and random forests

Furthermore, to screen the feature genes from DEAATGs, we trained two different machine-learning algorithms of lasso and RF.

**FIGURE 6:**
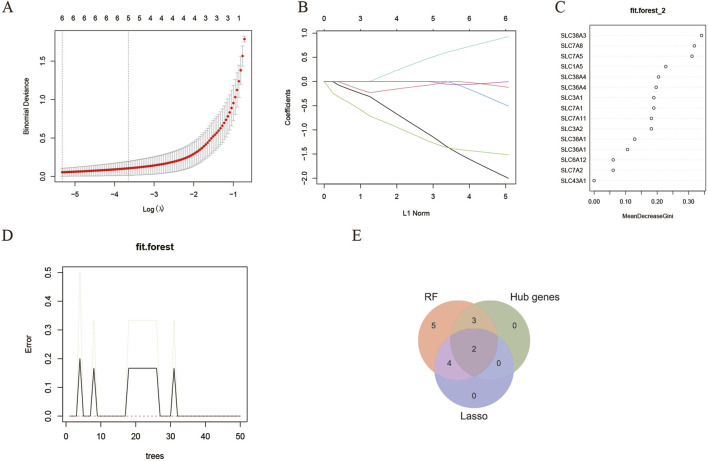
Identification of target genes. **(A, B)** The LASSO model; **(C, D)** The random forest model; **(E)** Identified target genes and venn diagram.

The LASSO regression is a machine-learning technique that assumes a linear relationship and incorporates an L1 regularization penalty. Initially, LASSO regression was conducted using 10-fold cross-validation to minimize the binomial deviance, resulting in an optimized λ value of 0.0325. Consequently, 6 genes with non-zero regression coefficients were chosen as feature genes for DEAATGs and included in the simplified LASSO regularization model (Figures 6A, B; Table 4).

**TABLE 4:** The feature genes of the LASSO and random forest model.

Machine learning algorithm	Feature genes
Lasso	SLC1A5, SLC7A5, SLC7A11, SLC36A1, SLC38A4, SLC7A1
Random Forest	SLC7A11, SLC7A8, SLC7A2, SLC6A12, SLC38A3, SLC3A2, SLC1A5, SLC38A1, SLC7A5, SLC36A1, SLC38A1, SLC36A4, SLC3A1, SLC7A1

The RF algorithm is a method for ensemble prediction. A random forest-supervised classification algorithm was employed to identify feature genes from DEAATGs, using the R randomforest package to create the RF models. The significance of each gene was assessed by computing the mean decrease in the Gini index (MeanDecreaseGini). Finally, there were 14 feature genes determined by the random forest model (Figures 6C, D; Table 4).

### Recognition of target genes and validation

We took an intersection of the three key gene sets screened by the PPI network (hub genes), lasso model, and random forest model and having two target genes, SLC7A5 and SLC1A5 (Figure 6E).

**FIGURE 7:**
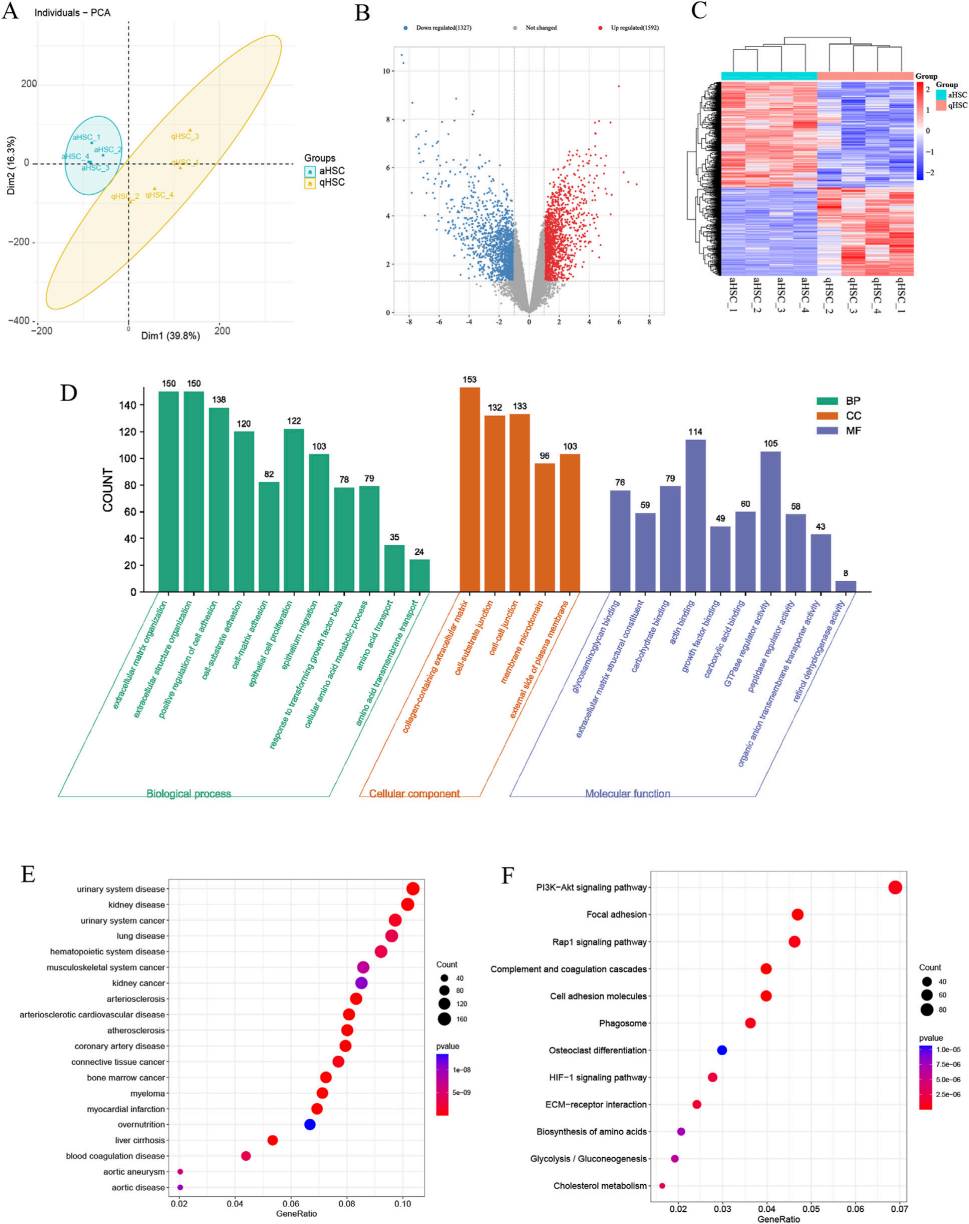
Validation of target genes. **(A)** The PCA plot of GES67664; **(B)** The volcano plot of the GSE67664; **(C)** The heatmap of DEGs in GSE67664; **(D)** GO enrichment; **(E)** DO enrichment; **(F)** KEGG signaling pathway enrichment.

**FIGURE 8:**
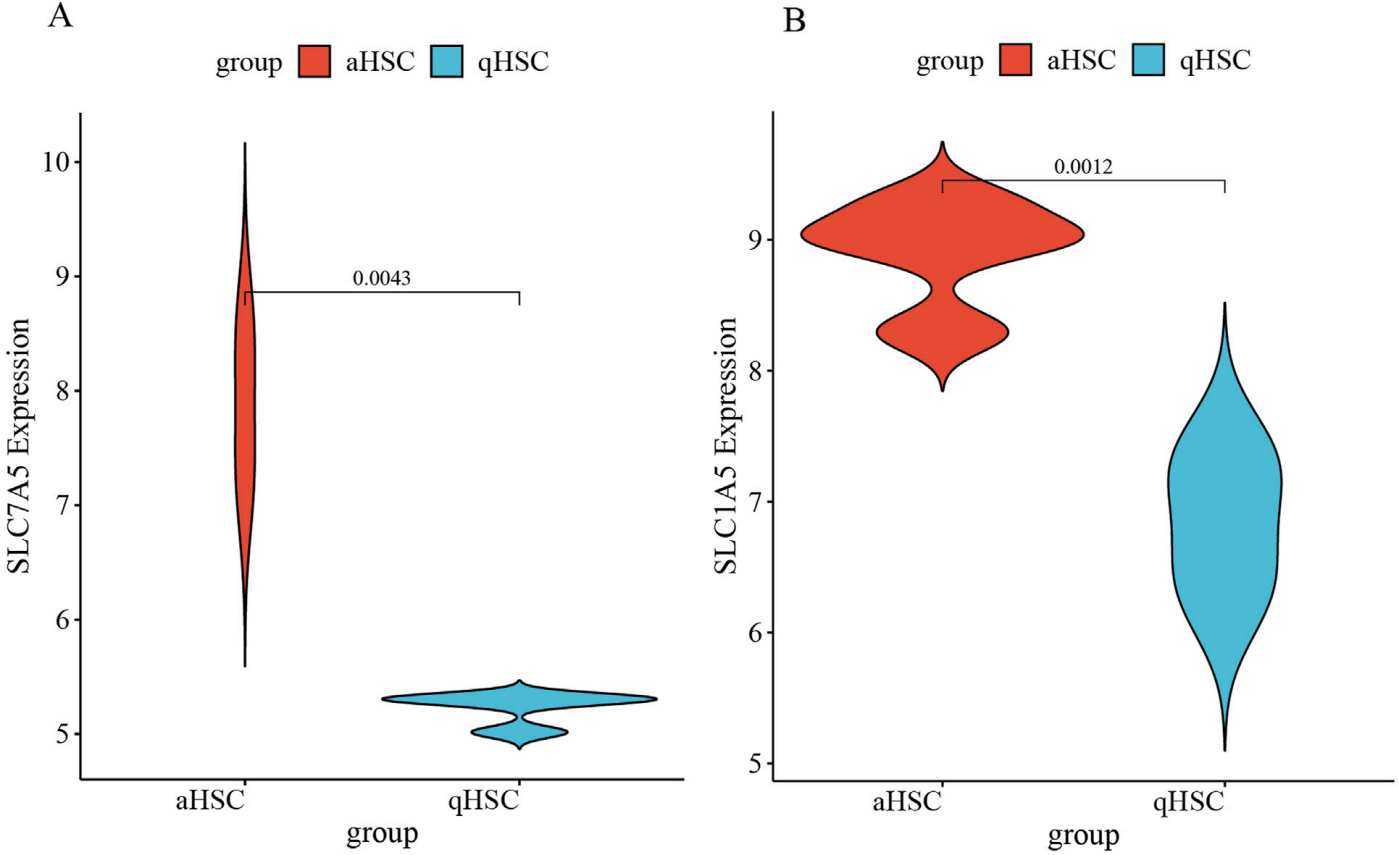
**(A)** The expression of SLC7A5 in GSE67664; **(B)** The expression of SLC1A5 in GSE67664.

The target genes were initially obtained from the training dataset GSE68000 and then validated using an independent validation dataset GSE67664. The basic information of the GSE67664 dataset is displayed in Figure 7A and Supplementary Figure S1. We identified 2,919 DEGs total, within which 1,592 were downregulated genes and 1,327 were upregulated genes (Figures 7B, C). Analysis of enrichment and signal pathways were executed for the DEGs. Similar enrichments of GO term were observed for all the clusters of BP, CC, and MF as well (Figure 7D). Details of the significant genes that participated in amino acid transmembrane transport and amino acid transport are given in Supplementary Figure S2. A description of DO disease enrichment and KEGG pathway enrichment is provided in Figures 7E, F. DEGs regulate liver cirrhosis, ECM−receptor interaction, and biosynthesis of amino acids. A study conducted using GSE67664 revealed that SLC7A5 and SLC1A5 of aHSC had significantly upregulated mRNA expression compared with the qHSC, which was consistent with the above results (Figures 8A, B). Therefore, our outcomes indicated that SLC7A5 and SLC1A5 might be the target genes for HSC activation.

### GSEA of target genes and GSVA

Since SLC7A5 and SLC1A5 may be pivotal in the amino acid transport of HSC and participate in the activation of HSC, and simultaneously the log2FC of them were maximum in the whole hub genes, we selected SLC7A5 and SLC1A5 for farther single-gene GSEA analysis separately via KEGG. The findings from the single-gene GSEA analysis aligned with the aforementioned results.

**FIGURE 9:**
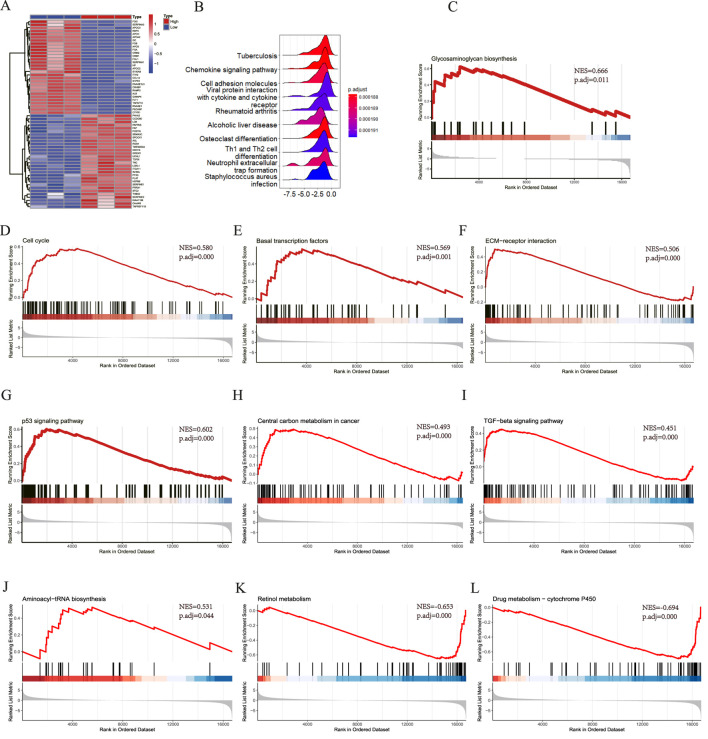
Single-gene GSEA of SLC7A5. **(A)** The top 30 genes of upregulated and downregulated DEGs in SLC7A5 high expression and low expression groups, the High means SLC7A5 high expression group, the Low means SLC7A5 low expression group; **(B)** The top 10 KEGG pathway ranked by enrichment score; **(C)** Glycosaminoglycan biosynthesis; **(D)** Cell cycle; **(E)** Basal transcription factors; **(F)** ECM−receptor interaction; **(G)** p53 signaling pathway; **(H)** Central carbon metabolism in cancer; **(I)** TGF−beta signaling pathway; **(J)** Aminoacyl−tRNA biosynthesis; **(K)** Retinol metabolism; **(L)** Drug metabolism − cytochrome P450.

**FIGURE 10:**
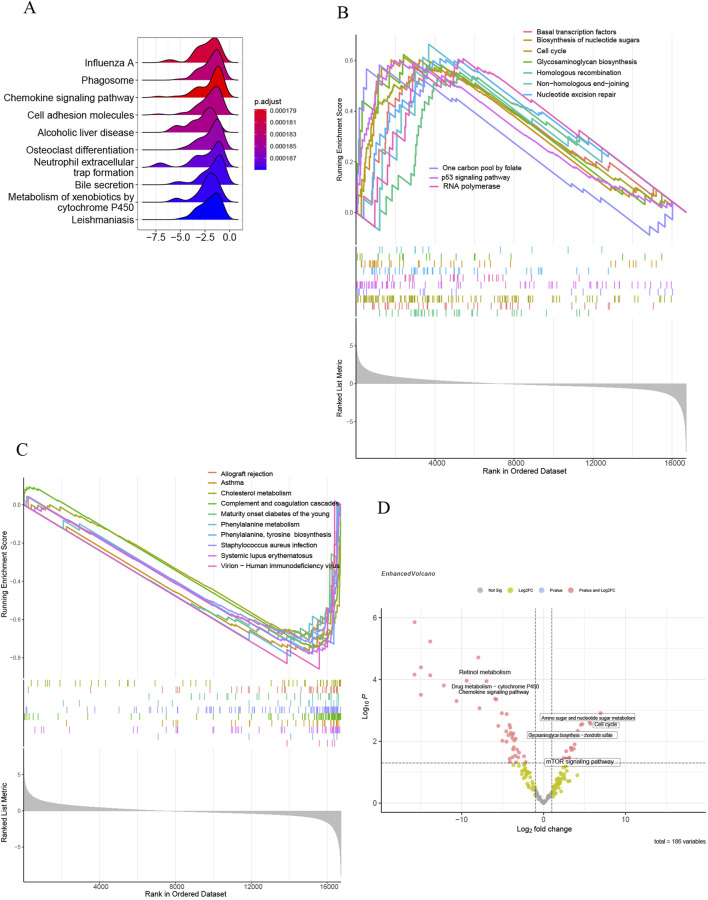
Single-gene GSEA of SLC1A5 and GSVA of GSE68000. **(A)** The top 10 KEGG pathways ranked by enrichment score; **(B)** The top 10 upregulated KEGG pathways ranked by NES; **(C)** The top 10 downregulated KEGG pathways ranked by NES; **(D)** The volcano plot of GSVA in GSE68000.

All samples were categorized into groups with high and low SLC7A5 expression according to mRNA levels. Differential expression evaluation was performed within these two groups, and a heatmap of the top 30 upregulated and downregulated differential expression genes is shown in Figure 9A. As Figures 9B–L shows, besides alcoholic liver disease, chemokine signaling pathway and cell adhesion molecules, SLC7A5 were still positively related to the glycosaminoglycan biosynthesis, cell cycle, basal transcription factors, ECM−receptor interaction, TGF−β signaling pathway, aminoacyl−tRNA biosynthesis. Inversely, SLC7A5 was negatively correlated with the retinol metabolism and Drug metabolism−cytochrome P450. Moreover, the outcomes of the SLC1A5 single-gene GSEA analysis were similar to the above, as Figures 10A–C shows that SLC1A5 was positively associated with glycosaminoglycan biosynthesis, biosynthesis of nucleotide sugars, cell cycle, and alcoholic liver disease.

Correspondingly, the results of GSVA analysis illustrated that the activated HSC group was closely correlated with the upregulation of the mTOR signaling pathway, cell cycle, and biosynthesis. Oppositely, the activation of HSC was negatively associated with retinol metabolism (Figure 10D).

### Correlation analysis with common key biomarkers of HSC activation

**FIGURE 11:**
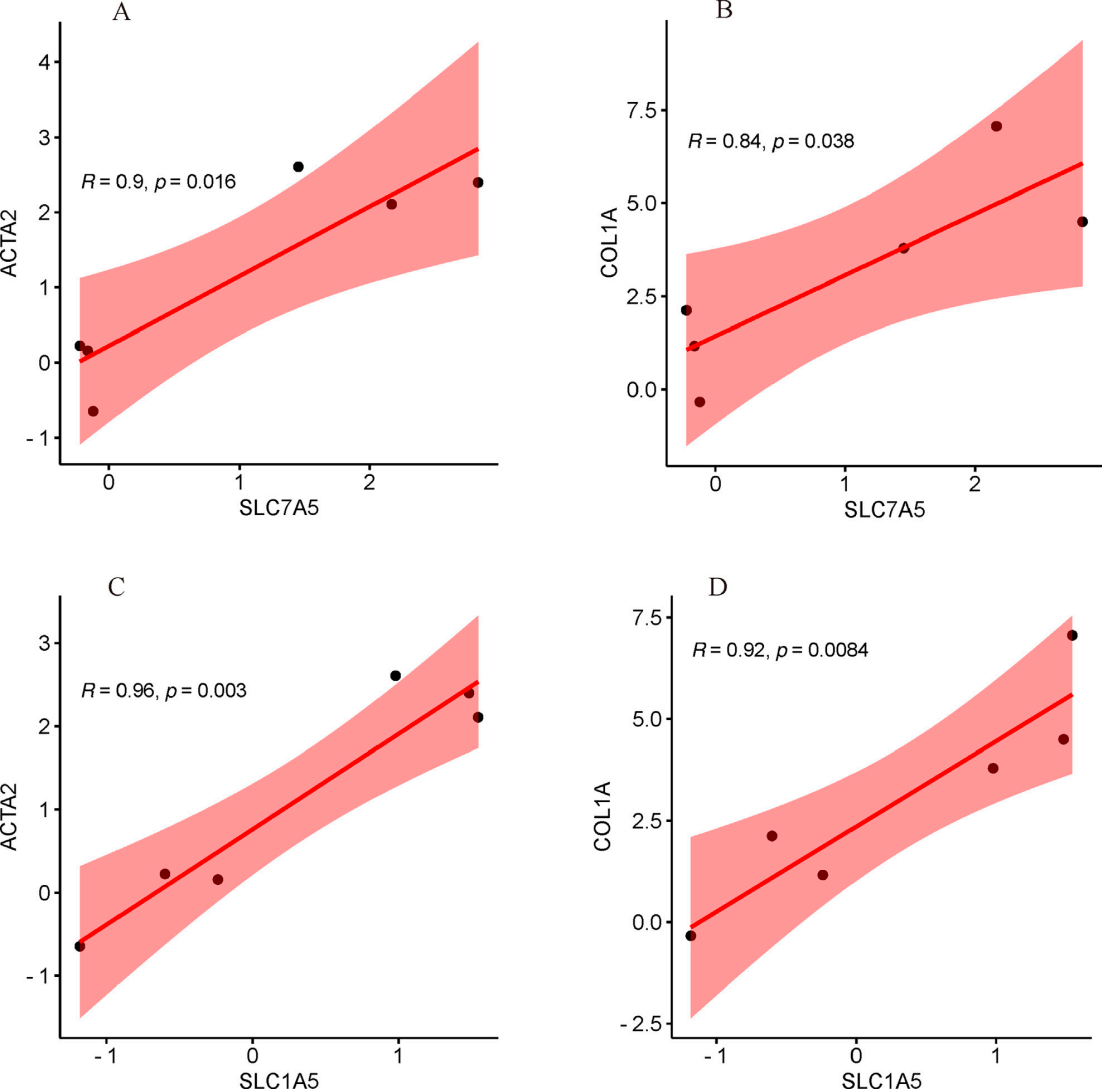
Correlation analysis with common key markers of activated HSC. **(A)** The correlation between SLC7A5 and ACTA2(α-SMA); **(B)** The correlation between SLC7A5 and COL1A; **(C)** The correlation between SLC1A5 and ACTA2(α-SMA); **(D)** The correlation between SLC1A5 and COL1A.

As illustrated in Figures 11A–D, the mRNA expression of both SLC7A5 and SLC1A5 were significantly positively correlated with ACTA2(α-SMA) and COL1A, indicating that SLC7A5 and SLC1A5 may associate with the activation of HSC closely.

### Subcellular localization and relationship with immune checkpoint

The biological functions of a particular protein are mainly associated with its different subcellular localizations. The subcellular localization of SLC7A5 and SLC1A5 was plasma membrane, predicted via Cell-PLoc 2.0. This characteristic suggests that SLC7A5 and SLC1A5 may be essential for the transmembrane transport of amino acids.

**FIGURE 12:**
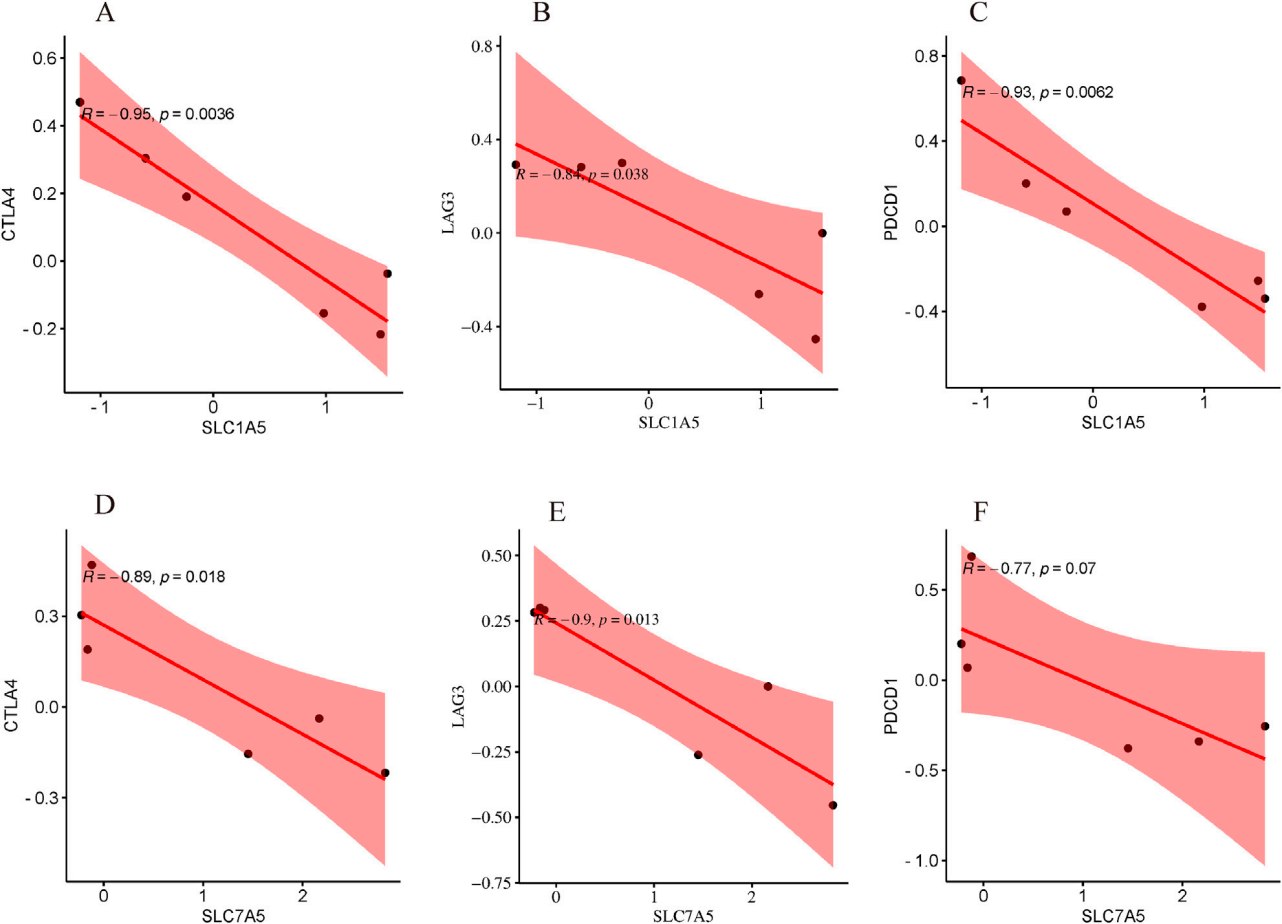
Correlation analysis with immune checkpoint. **(A)** The correlation between SLC1A5 and CTLA4; **(B)** The correlation between SLC1A5 and LAG3; **(C)** The correlation between SLC1A5 and PDCD1; **(D)** The correlation between SLC7A5 and CTLA4; **(E)** The correlation between SLC7A5 and LAG3; **(F)** The correlation between SLC7A5 and PDCD1.

In a physiological status, immune checkpoints are necessary for immunologic tolerance, to block autoimmunity, and to preserve normal tissues from immune injury (Wang H. et al., 2019). As presented in Figures 12A–F, there was a significant negative correlation within SLC7A5 conventional immune checkpoints containing LAG3 and CTLA4; meanwhile, a significant negative correlation between SLC1A5 and PDCD1, LAG3, and CTLA4 was verified. Their results revealed that SLC7A5 and SLC1A5 might further occupy an essential position in the immune response.

### mRNA–miRNA–lncRNA ceRNA network

**FIGURE 13:**
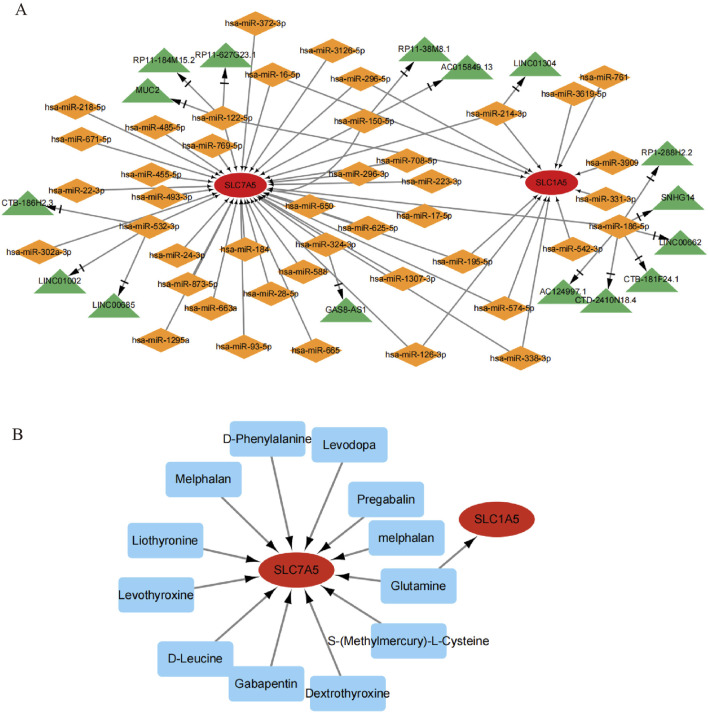
The ceRNA network and drug-gene interaction network of target genes; **(A)** The ceRNA network of target genes, the ellipse means mRNA of target genes, the diamond means miRNAs, and the triangle means lncRNAs; **(B)** The drug-gene interaction network of target genes, the ellipse means target genes and the rectangle means drugs.

It is well-known that miRNAs are involved in regulating gene expression at the post-transcriptional level. Moreover, the biological function of Long Non-coding RNA (lncRNA) cannot be ignored, although it does not participate in encoding proteins. The lncRNA can modulate the expression of mRNA through their interactions with miRNA in the mRNA-miRNA-lncRNA ceRNA network, and disturbance of these networks may impact diseases. Decades of miRNAs predicted to target 3′-UTR of the SLC7A5 and SLC1A5 were identified by using three miRNA target-predicted databases. The interactional miRNA of lncRNA was searched and analyzed via the spongeScan database. Finally, a ceRNA network (containing 2 target genes, 16 LncRNAs, and 42 miRNAs) was identified in Figure 13A.

### Drug-gene interplay network

The DGIdb and DrugBank databases were used to explore drug-gene interactions and identify existing or potential pharmaceuticals. Targeting SLC7A5 and SLC1A5 could provide a specific treatment strategy. The complete drug-gene interaction network for these genes is shown in Figure 13B. In total, 12 potential therapeutic drugs were identified.

## Discussion

Liver fibrosis is a common hepatic disease posing a critical threat to human health. The molecular mechanisms of liver fibrosis are complex, involving multiple molecular and signal pathway mechanisms. Although recent treatments of liver fibrosis have slight improvement and effectiveness, how to more effectively prevent and reverse it remains a significant challenge worldwide as there are still great unknowns in its direct genesis. Since HSC is the most relevant mesenchymal cell contributing to liver fibrosis, most suggested anti-fibrotic treatments were based on the molecular mechanisms related to HSC activation (Malaguarnera et al., 2015). According to AAT, there is a promising research potential in liver fibrosis that parallels that of malignant tumors. This study was conducted to identify and characterize potential biomarkers for aHSC by examining amino acid transport-related genes through bioinformatics approaches. The focus was particularly on the accumulation of ECM and myofibroblasts, to provide novel perspectives on the underlying etiopathogenesis and possible treatments for liver fibrosis.

In this study, we identified 3,775 DEGs within the aHSC mRNA-expression profile, and functional enrichment analyses of the DEGs were performed. Amino acid metabolism is essential for the rapid metabolic reprogramming of classically activated HSC (Trivedi et al., 2021). Our study has shown that the DEGs mostly took part in the transport and biosynthesis of amino acids according to the function enrichment analyses. These results inspired us to execute in-depth research on the correlation between amino acid transport and HSC activation. Then, a total of 15 DEAATGs were identified by crossing DEGs with genes related to amino acid transport, with 8 being upregulated and 7 downregulated. and utilized for functional gene analysis. All of the DEAATGs belong to the solute carrier family (SLC). The SLC family, a superfamily located on the eukaryotic plasma membrane, regulates the uptake and outflow of multiple solutes, such as amino acids, sugars, and drugs (Cropp et al., 2008). The SLC family has many crucial functions in eukaryote biology and correlates with cellular protein and nucleotide biosynthesis, especially those cells in high demand for substances (Coothankandaswamy et al., 2016). As shown in the GO cluster, the DEAATGs mostly participated in the transport and metabolic process of amino acids. The DEAATGs are mainly involved in metabolism and signaling pathways according to the KEGG pathway, likely central carbon metabolism, protein digestion, absorption, and the mTOR signaling pathway. The Wnt signaling pathway was proven to be associated with central carbon metabolism and suppression of HSC activation through mediating the biosynthesis of glutamine synthetase and reducing ammonia levels (Russell and Monga, 2018). Additionally, the mTOR pathway, playing a central role in cell metabolism and growth (Li et al., 2019), is closely correlated with HSC activation (Zhang et al., 2019). Moreover, the result of DO further corroborates the above. These outcomes of gene function analysis for DEAATGs revealed that, to some extent, the SLC family might drive adjustments of amino acid metabolism and function to promote activation of HSC.

The interaction associations between DEAATGs-encoded proteins were investigated using the PPI and its modules. Subsequently, five hub genes were identified from it, namely, SLC7A5, SLC1A5, SLC7A8, SLC3A1, and SLC3A2. While proteins are vital for biological functions and the PPI network is crucial within the body, it does not, in isolation, offer a comprehensive representation of the intricate biological regulatory network (He et al., 2023). To address the limitations inherent in a singular model, we utilized additional algorithms to concurrently identify feature genes. Recently, machine learning algorithms have been extensively applied in bioinformatics analyses to filter diagnostic biomarkers and construct prognostic models (Fabris et al., 2018). Consequently, the LASSO regression and RF algorithm were employed to further assess the relative importance of feature genes, thereby facilitating dimensionality reduction and feature selection. To mitigate the risk of overfitting or selection bias, LASSO regression was validated using 10-fold cross-validation, in conjunction with the RF algorithm, to identify the signature genes. Finally, SLC7A5 and SLC1A5 genes are identified as targets based on the overlap between hub and feature gene sets. The validation dataset GSE67664 confirmed the mRNA expression of SLC7A5 and SLC1A5. Although these methods yielded more accurate results compared to those based solely on PPI networks, they were not without limitations. Notably, the use of a small dataset increases the risk of the model becoming either overfitted or underfitted during training. Consequently, future research employing larger sample sizes is necessary to validate these findings.

Previous research has demonstrated that the activation of HSC can be inhibited by modulating the expression of critical genes involved in metabolic reprogramming. During liver injury, aHSC exhibits increased cell proliferation, fibrogenesis, contractility, chemotaxis, and cytokine release. The activation and functionality of HSC are contingent upon metabolic alterations. Consequently, supporting the energy metabolism of HSC may serve as a potential strategy for prevention (Bae et al., 2022). During the activation of HSC, there is an increased demand for essential amino acids, such as leucine, as well as an enhanced uptake of glutamine. The transmembrane protein SLC7A5 is responsible for facilitating the transport of essential amino acids into cells, while SLC1A5 plays a significant role in the uptake of glutamine. In addition to facilitating cell proliferation, glutamine plays a crucial role in phagocytosis, the synthesis and secretion of pro-inflammatory cytokines, and antigen presentation. Notably, macrophages overexpressing HMBOX1 exhibited a significant reduction in glutamine concentrations, accompanied by the downregulation of the glutamine transporter SLC1A5. Furthermore, the HMBOX1/SLC1A5-mediated reduction in glutamine uptake may represent a potential mechanism underlying the protective effects of HMBOX1 in liver inflammation (Jiang et al., 2023). Consequently, targeting the transport mechanisms of these amino acids presents a potential novel therapeutic strategy.

There is a crucial role for SLC7A5 in promoting cell growth and proliferation (Chai et al., 2022). In addition to nutrition, stress, and energy state, mTOR also plays a key role in intracellular signaling (Zhang et al., 2022). SLC7A5 transports amino acids including leucine as a result of activation of the AKT/mTOR signaling pathway (Chen et al., 2022). SLC7A5 knockdown reduced mTOR pathway activity and suppressed the proliferation and metastasis of tumor cells (Li et al., 2021). Recent reports suggest that cancer cells may shift their energy source from glucose to glutamine and manifest as a glutamine-dependent phenotype via metabolic reprogramming (Sikder et al., 2020). S. Tanaka reported that downregulated SLC1A5 expression could lead to the diminished activity of mTORC1, resulting in weakened cell proliferation (Tanaka et al., 2018). In the experimental model of hepatic fibrosis, following hepatic cell injury, activation of the mTOR pathway in mesenchymal cells enhanced the wound healing response. In summary, overactivation of mTOR within the mesenchymal compartment exacerbated liver fibrosis induced by CCl4 (Shan et al., 2016). AKT and mTOR, which are pivotal components of the PI3K pathway, have the potential to regulate the activation of HSC and the progression of liver fibrosis (Huang et al., 2023). Furthermore, liver fibrosis can be mitigated through AMPK phosphorylation and inhibition of mTOR-dependent signaling cascades (Wang et al., 2020). In this study, we observed that the upregulation of SLC7A5 and SLC1A5 is closely linked to enhanced activity of the mTOR pathway, corroborating previous research findings. We hypothesize that the transporters SLC7A5 and SLC1A5 could serve as novel biomarkers for the initial activation of HSC by modulating the activity of the mTOR signaling pathway.

During the progression of liver fibrosis, the TGF-β signaling pathway plays a crucial role (Peng W. et al., 2022). Inhibition of this pathway may lead to a reduction in hepatic fibrosis (Kundu et al., 2023). Various serum markers indicative of ECM components are employed to evaluate the progression of liver fibrosis, a condition marked by the excessive accumulation of ECM (Wu et al., 2017). In the human body, retinol is sequestered in HSCs, which are pivotal in the fibrogenic processes of the liver (Saki et al., 2020). To investigate the relationship between the target genes and principal markers of liver fibrosis, we conducted a single-gene GSEA analysis. The single-gene GSEA utilizing the KEGG pathway for SLC7A5 and SLC1A5 individually revealed a noteworthy positive correlation between the upregulation of SLC7A5 and the ECM-receptor interaction, cell cycle, and TGF-β signaling pathways. Conversely, this upregulation exhibited a significant negative association with retinol metabolism. There were similar results in single-gene GSEA analysis of SLC1A5, and both SLC1A5 and SLC7A5 were closely related to alcoholic liver disease, which is known as a common genesis of liver fibrosis around the world (Heo et al., 2019). The pieces of evidence that the TGF-βsignaling pathway places a central position during every step of the development of HSC activation and hepatocarcinogenesis were strong. Treatments aimed at depressing the TGF-βsignaling pathway have conspicuously prevented liver fibrosis *in vivo* models (Liu et al., 2019). During HSC activation, the decreased expression of retinol acyltransferase (LRAT) may lead to a reduction of retinol significantly (Zhang et al., 2021), and the deficiency of retinol is known to promote fibrosis development of the liver (Aguilar et al., 2009). Consequently, utilizing single-gene GSEA, we identified that the alteration of the target genes resulted in a consistent modulation of several liver fibrosis markers. This finding further substantiates the pivotal role of the target genes in the activation of hepatic stellate cells from an alternative perspective.

Additionally, due to the limited number of data samples, constructing a nomogram and ROC curve for predicting HSC activation based on the target gene was challenging, as the area under the curve (AUC) equaled 1, indicating overfitting. Consequently, we conducted scatter plot analyses of the correlation coefficients and performed statistical tests between the target genes and conventional diagnostic markers of liver fibrosis, specifically α-SMA and COL1A. The expression patterns of the target genes were found to be completely consistent with those of the traditional markers, according to the results. Ultimately, these results show a possibly vital role in the overexpression of SLC7A5 and SLC1A5 in the activation of HSC.

The numerous intrahepatic immunocytes play an important role in maintaining hepatic homeostasis and are the essential mechanisms for many liver illnesses (Liang et al., 2020a). Recently, many studies have indicated that various intrahepatic immunocyte subcategories (containing B cells, T-cells, macrophages, and neutrophils) performed a crucial position in the progression of liver inflammation and fibrosis (Liang et al., 2020b). An anomalous immune cell activation could lead to a dysfunctional immune microenvironment in the liver, inducing HSC to differentiate into myofibroblast-like cells and drive liver fibrosis (Ikeno et al., 2020). Immune homeostasis is crucially associated with the regulation of immune checkpoints under normal physiological conditions and inhibits irregular autoimmune damage to healthy tissues (Liu et al., 2020). However, cancer cells can escape immune attack via the expression of immune checkpoints (Tu et al., 2020). Then we speculate that immune checkpoints might correlate with HSC activation and liver fibrosis and found that SLC1A5 was significantly negatively associated with CTLA4, LAG3, and PDCD1; similarly, SLC7A5 was significantly negatively associated with CTLA4 and LAG3. CTLA4 delivers an inhibitory signal to the T-cell as a T-cell transmembrane receptor, and the antagonist of CTLA4 activates the immune system and prevents T-cell exhaustion (Welsh et al., 2019). The proliferation, activation, and effector function of CD4^+^ and CD8^+^ T-cells were negatively modulated by LAG3 expression, which has also been reported to regulate autoimmunity (Wang J. et al., 2019). It was found in clinical studies that anti-PD-1 and anti-CTLA-4 monotherapy could lead to a higher incidence of acute liver injury, and anti-PD-L1 antibodies such as atezolizumab may promote fast progression of liver fibrosis, triggered by acute intrahepatic immunocytes infiltration of CD4^+^ and CD8^+^ T-cells principally (Honma et al., 2021). Jing Xu reported that SLC7A5 performed as a checkpoint for the activation of T-cells through the mTOR pathway (Xu et al., 2020). Taken together, we automatically hypothesized that upregulated SLC7A5 and SLC1A5 might contribute to the development of liver fibrosis by suppressing the expression of the immune checkpoint and inducing the anomalous immunocyte activation intrahepatic. Undeniably, our knowledge of this perspective has been extraordinarily limited recently, and more evidence is imminently needed to support this hypothesis in the future.

Finally, to provide drug treatment strategies for HSC activation, we further identified 12 possible therapeutic drugs targeting SLC7A5, and one of them may have a potential therapeutical effect for SLC1A5 too. Moreover, a ceRNA network was constructed to demonstrate a potentially interactive regulation in HSC activation, which will be helpful for future studies on the post-transcriptional regulatory mechanism.

Furthermore, as far as we know, this research is the first to propose that SLC7A5 and SLC1A5 might be involved in HSC activation. Nevertheless, our research is subject to certain limitations. Notably, the GEO database offers a limited number of datasets and samples on the mRNA expression profiles of activated HSC *in vitro*. Moreover, the cell culture model of activated HSC may not entirely correspond with *in vivo* data. It is noteworthy that the expression of the target genes exhibited consistent patterns across two independent datasets, thereby minimizing the potential for batch-to-batch variation and strengthening the validity of our study. Furthermore, further research involving larger sample sizes is required to substantiate the diagnostic efficacy of these target genes in the activation of HSC, and future *in vivo* experiments are planned to substantiate our findings. Additionally, further investigation is required to elucidate the regulatory mechanisms of SLC7A5 and SLC1A5 within the context of HSC activation.

## Conclusions

We identified two target genes, SLC7A5 and SLC1A5, from amino acid transport-related genes, which mainly participated in the ECM−receptor interaction, cell cycle, TGF−β signaling pathway, and retinol metabolism, and also closely correlated with the familiar biomarkers of liver fibrosis and immune checkpoints. Therefore, SLC7A5 and SLC1A5, with their relevant molecules, might be potentially vital biomarkers for HSC activation to some extent. These results would supply a novel insight into the pathogenesis and therapeutical approaches of liver fibrosis.

## Data Availability

Publicly available datasets were analyzed in this study. This data can be found here: https://www.ncbi.nlm.nih.gov/geo/query/acc.cgi?acc=GSE67664; https://www.ncbi.nlm.nih.gov/geo/query/acc.cgi?acc=GSE68000.
