# Supplementary figures and images for: Identification of potential biomarkers from amino acid transporter in the activation of hepatic stellate cells via bioinformatics
Source: Front Genet. 2024 Dec 4;15:1499915. doi: 10.3389/fgene.2024.1499915 (PMC11652522; doi:10.3389/fgene.2024.1499915)

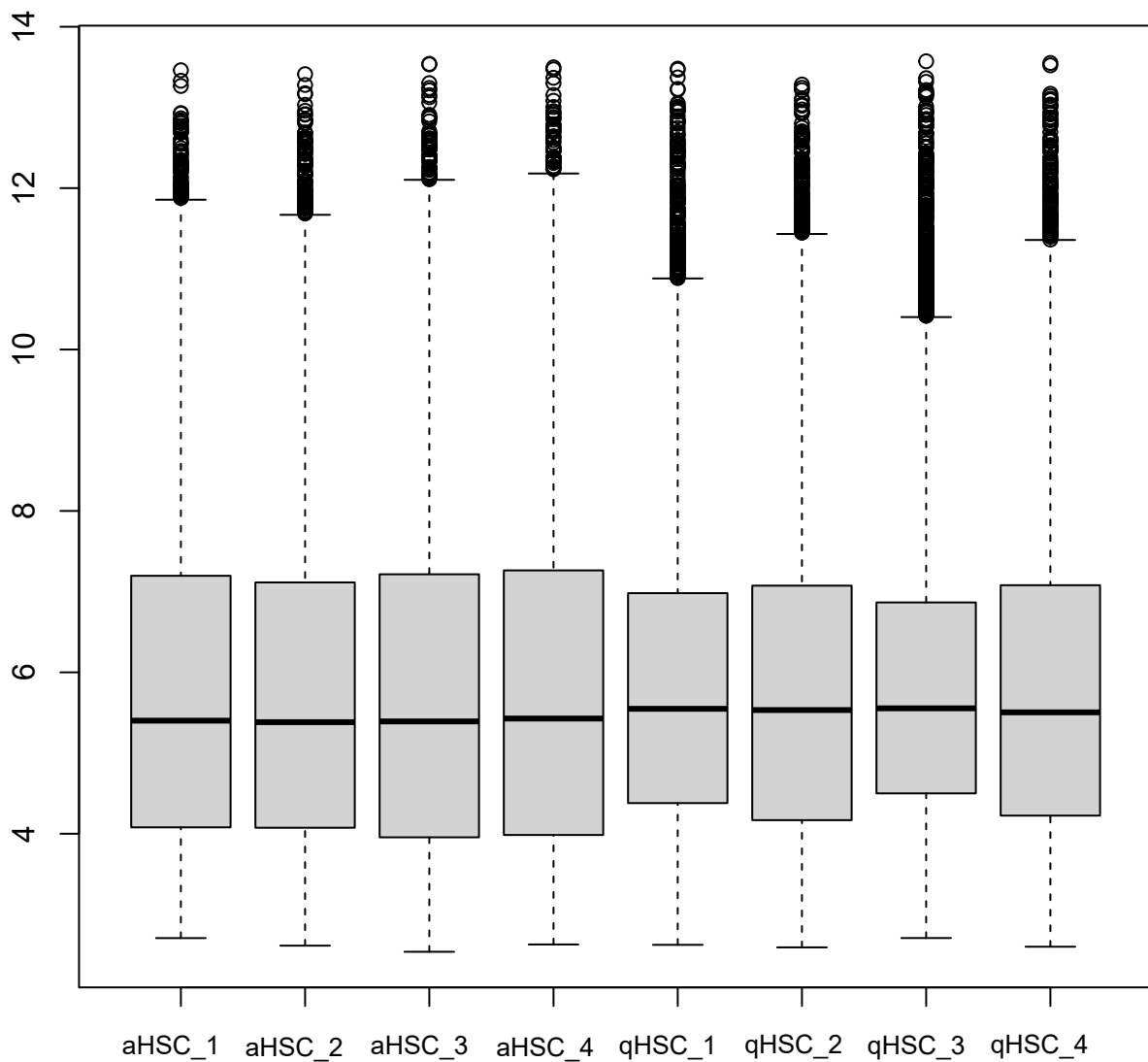

Supplement: Supplementary file 1 [file Image2.PDF]

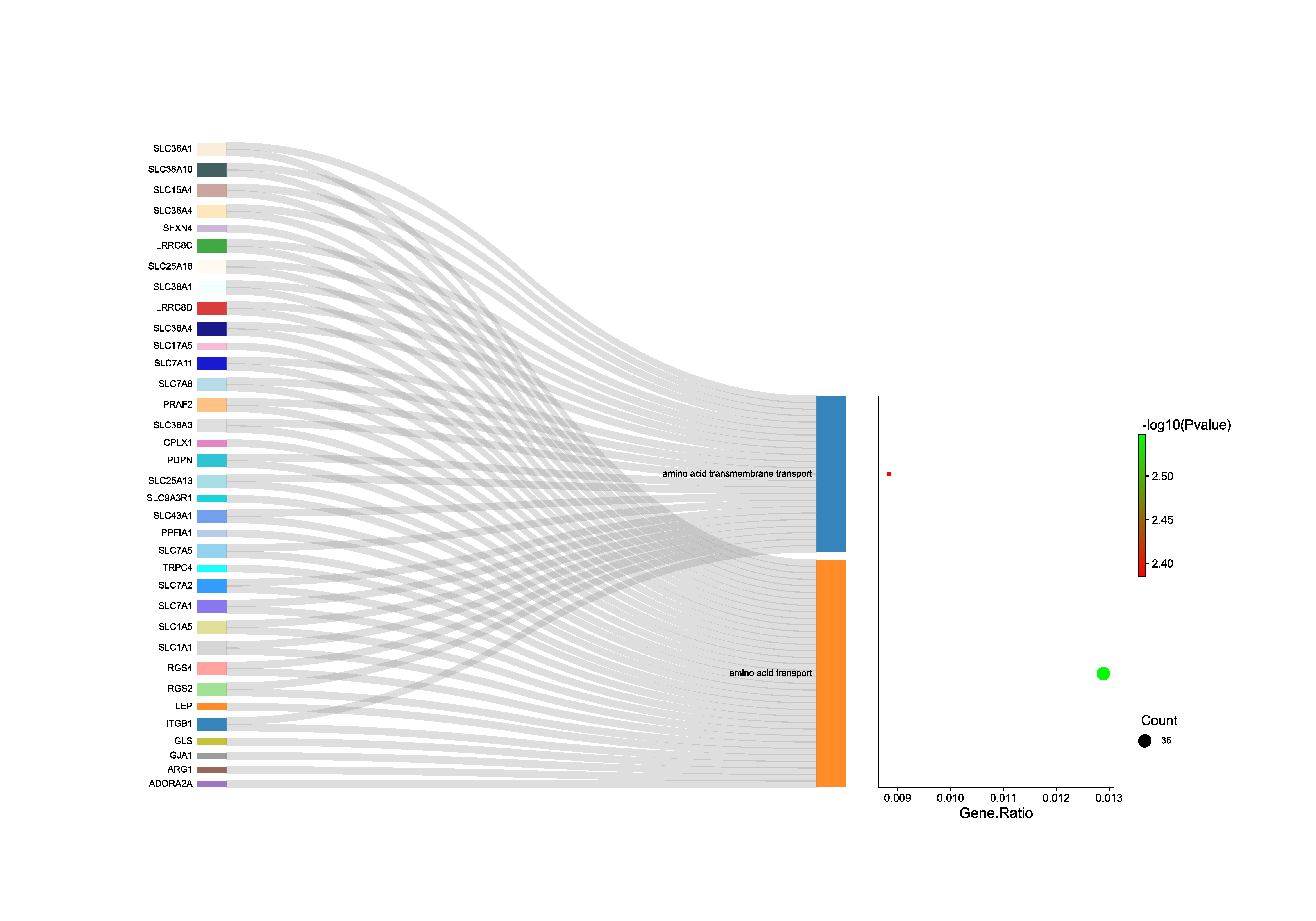

Supplement: Supplementary file 3 [file Image1.PNG]
