# Supplementary material for: Identification of potential biomarkers from amino acid transporter in the activation of hepatic stellate cells via bioinformatics
Source: Front Genet. 2024 Dec 4;15:1499915. doi: 10.3389/fgene.2024.1499915 (PMC11652522; doi:10.3389/fgene.2024.1499915)
Supplement: Supplementary file 2 [file Table1.DOC]

**TABLE 1** Details of the GEO data.

| Dataset | Platform | Number of samples (activated / quiescent HSC, subjects) |
| --- | --- | --- |
| GSE68000 | GPL13667  [HG-U219] Affymetrix Human Genome U219 Array | 11(3/3,6) |
| GSE67664 | GPL19099  [HG-U219] Affymetrix Human Genome U219 Array | 13(4/4,8) |

GEO, Gene Expression Omnibus.

**TABLE 2** The DEAATGs of GSE68000

| Regulation | DEAATGs |
| --- | --- |
| Upregulated (n = 8) | SLC1A5、SLC38A1、SLC7A5、SLC36A1、SLC38A1、SLC36A4、SLC3A1、SLC7A1 |
| Downregulated (n = 7) | SLC7A11、SLC7A8、SLC7A2、SLC6A12、SLC43A1、SLC38A3、SLC3A2 |

DEAATGs, differently expressed amino acid transport-related genes.

**TABLE 3** The top 5 hub genes

| Genes | MCC | MNC | Degree | Stress | Betweenness | Log_2_FC |
| --- | --- | --- | --- | --- | --- | --- |
| SLC7A5 | 6 | 3 | 5 | 22 | 10.3 | 2.143191791 |
| SLC1A5 | 8 | 5 | 5 | 16 | 7.5 | 2.382896913 |
| SLC7A8 | 4 | 2 | 4 | 16 | 5.3 | -1.580877908 |
| SLC3A1 | 3 | 2 | 3 | 4 | 1.3 | 1.550995367 |
| SLC3A2 | 4 | 2 | 4 | 16 | 5.3 | -1.237840961 |

MCC, maximal clique centrality; MNC, maximum neighborhood component.

**TABLE 4** The feature genes of lasso and random forest model.

| Machine learning algorithm | Feature genes |
| --- | --- |
| Lasso | SLC1A5、SLC7A5、SLC7A11、SLC36A1、SLC38A4、SLC7A1 |
| Random Forest | SLC7A11、SLC7A8、SLC7A2、SLC6A12、SLC38A3、SLC3A2、SLC1A5、SLC38A1、SLC7A5、SLC36A1、SLC38A1、SLC36A4、SLC3A1、SLC7A1 |
